# Analgesia Nociception Index: Heart Rate Variability Analysis of Emotional Status

**DOI:** 10.7759/cureus.4365

**Published:** 2019-04-02

**Authors:** Ruslan Abdullayev, Ercan Yildirim, Bulent Celik, Leyla Topcu Sarica

**Affiliations:** 1 Anesthesiology and Reanimation, Marmara University School of Medicine, İstanbul, TUR; 2 Anesthesiology and Reanimation, Gazi University School of Medicine, Ankara, TUR; 3 Statistics, Gazi University Faculty of Sciences, Ankara, TUR; 4 Anesthesiology and Reanimation, Adiyaman University Educational and Research Hospital, Adiyaman, TUR

**Keywords:** analgesia/nociception index, heart rate variability, ani, music, pain, analgesia

## Abstract

Background

Analgesia nociception index (ANI) has been developed for real-time pain measurement during a surgical procedure under general anesthesia. The index is based on heart rate variability and constitutes a measure of parasympathetic tone. In this paper, we hypothesized that this index could be used as a tool to investigate the process of emotional regulation of a human subject.

Materials and methods

Twenty adult volunteers were recruited for the study, wherein ANI response to the emotional stimulus was evaluated. An emotional stimulus was obtained through a 60-second music sound record from the song “Ala Gözlerini Sevdiğim Dilber,” performed by the Turkish rock band Badem. ANI measurements were obtained before the song presentation (T_pre_), at the end of the record presentation (T0), and each minute thereafter until the end of the five-minute observation (T1-T5).

Results

Twenty participants were investigated; 10 males and 10 females. The mean age of the participants was 17.0 ± 0.9 (min: 16, max: 20). ANI measurements were significantly lower in T0 and T3 compared with T_pre_ (*P* = 0.009). The differences between other values were not statistically significant.

Conclusion

ANI can be used for assessment of parasympathetic changes related to the emotional state of conscious patients.

## Introduction

Emotional stimuli activate the autonomic nervous system (ANS) to increase the physiological and psychological vigilance to cope with the incoming situation. ANS is composed of two distinct parts: the sympathetic and the parasympathetic nervous systems. ANS flexibility influences the way an individual pass from a state of alert in the case of emotional situation to a state of calm [1]. This is the capacity to regulate his emotions. This flexibility of the ANS mainly depends on the vagus nerve, which constitutes mainly the parasympathetic part of it [2-3].

Heart rate variability (HRV) is a commonly used noninvasive measure of autonomic control of the heart. Variations in heart rate (HR) above 0.15 Hz and centered at the respiratory frequency are mediated by changes in the parasympathetic outflow only, whereas lower frequency changes are mediated by both the parasympathetic and sympathetic outflow changes [4-5]. Fear and anxiety result in a decrease of the HRV high frequency (HF) content (between 0.15 and 0.5 Hz), indicating the decrease in the parasympathetic activity during unpleasant stimuli or emotion [6-8]. During surgery, HRV is correlated with the balance between the level of analgesia and the extent of the nociceptive stimulus [6]. Other studies have shown that stress and anxiety levels are correlated with HRV HF content [6,9-10].

Analgesia nociception index (ANI) was developed for pain evaluation during surgery under general anesthesia [11-12]. The index can be considered as a vagal tone index that is based on the ventilatory influence on the heart rate. It provides both qualitative and quantitative measurements of HRV.

ANI has been used as a tool to measure changes in the emotional status of healthy volunteers, where the negative emotional stimulus consisted of a negative emotion-eliciting video, and it was associated with a decreased ANI [13].

In this study, we used a music record of 60-second duration to create an emotional stimulus, and the hypothesis was that the emotional change created by the music would be reflected on ANI.

## Materials and methods

A. HRV analysis

The electrocardiography (ECG) signal is obtained from the patient using classical ECG electrodes and digitized at a sampling rate of 250 Hz. The RR series is built as the time evolution of the time intervals between two successive R waves of the ECG (RR value). A previously described R wave detection algorithm are used for R peak detection [14]. RR interval series is filtered in real time using an original non-linear filtering algorithm, because of disruption caused by perturbations like ectopic beats or electrode motion [15]. This algorithm is based on the morphological analysis of the RR series, and detects the disturbed area and replaces the erroneous samples with the most probable ones. A linear interpolation algorithm was used to re-sample the filtered RR series at 8 Hz. After mean centering, the 8 Hz resampled RR series are normalized, using the vectorial norm of the RR series over 64 seconds. The norm values (S) is computed as follows:

$$S = \sqrt{\sum_{i=1}^{N}{(RR_{i})}^{2}}$$

Then, each RR sample is divided by the norm value S:

 RR’i = RRi / S

The mean-centered and normalized RR series are then band-pass filtered from 0.15 to 0.5 Hz using a wavelet transform-based numerical filter providing RRHF, and hence, only HF variations are kept. The amplitude of the normalized and filtered RR series ranges between 0 and 0.2 (normalized unit, n.u.) [13].

**Figure 1:**
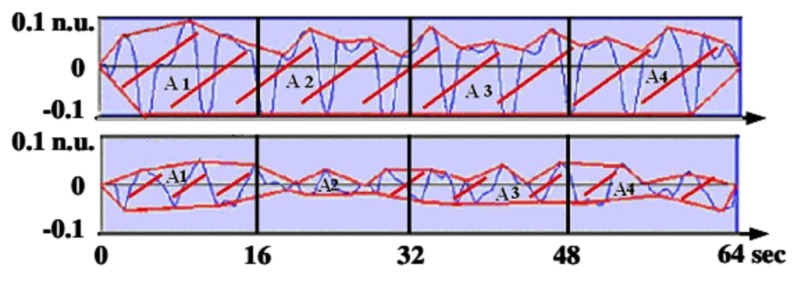
Normalized and filtered RR series in two different states of analgesia/nociception balance during general anesthesia A1, A2, A3, and A4 are the areas measuring the respiratory influence in the RR series; upper panel: adequate analgesia; lower panel: light analgesia leading to an increase of heart rate and blood pressure

When the parasympathetic tone is present, each respiratory cycle influences the RR series, causing a brief decrease in heart period. This corresponds to a short increase of HR, which is known as respiratory arrhythmia (Figure 1, upper panel). When the parasympathetic tone decreases, the influence of each respiratory cycle is dampened (Figure 1, lower panel). This is how the area of respiratory influence in the RR series can be used to assess parasympathetic tone activity, and thus, analgesia/nociception balance. When nociception grows, or analgesia wears off, sympathetic activity increases, and the parasympathetic tone decreases [13].

In order to transform this qualitative observation into quantitative information, a graphical index by computing the area under the RRHF series curve, as shown in Figure 1, was developed [13,16]. Local minima and maxima are detected and the upper and lower envelopes are plotted by connecting the local maxima together and the local minima as well. This allows obtaining an index which is independent of the respiratory frequency changes.

The 64-second moving window is then divided into four sub-windows of 16 seconds to increase the sensitivity of the method. The areas between the lower and upper envelopes are then measured in the four sub-windows [13].

ANI is then computed to express a fraction of the total window surface, leading to a measure between 0 and 100.

ANI = 100 x [α x AUCmin + β] / 12.8

In order to keep the coherence between the visual effect of respiratory influence on RR series and the quantitative measurement of ANI, α and β values have been empirically determined in a general anesthesia data set of more than 100 patients (α: 5.1, β: 1.2) [13].

B. Monitoring device

**Figure 2:**
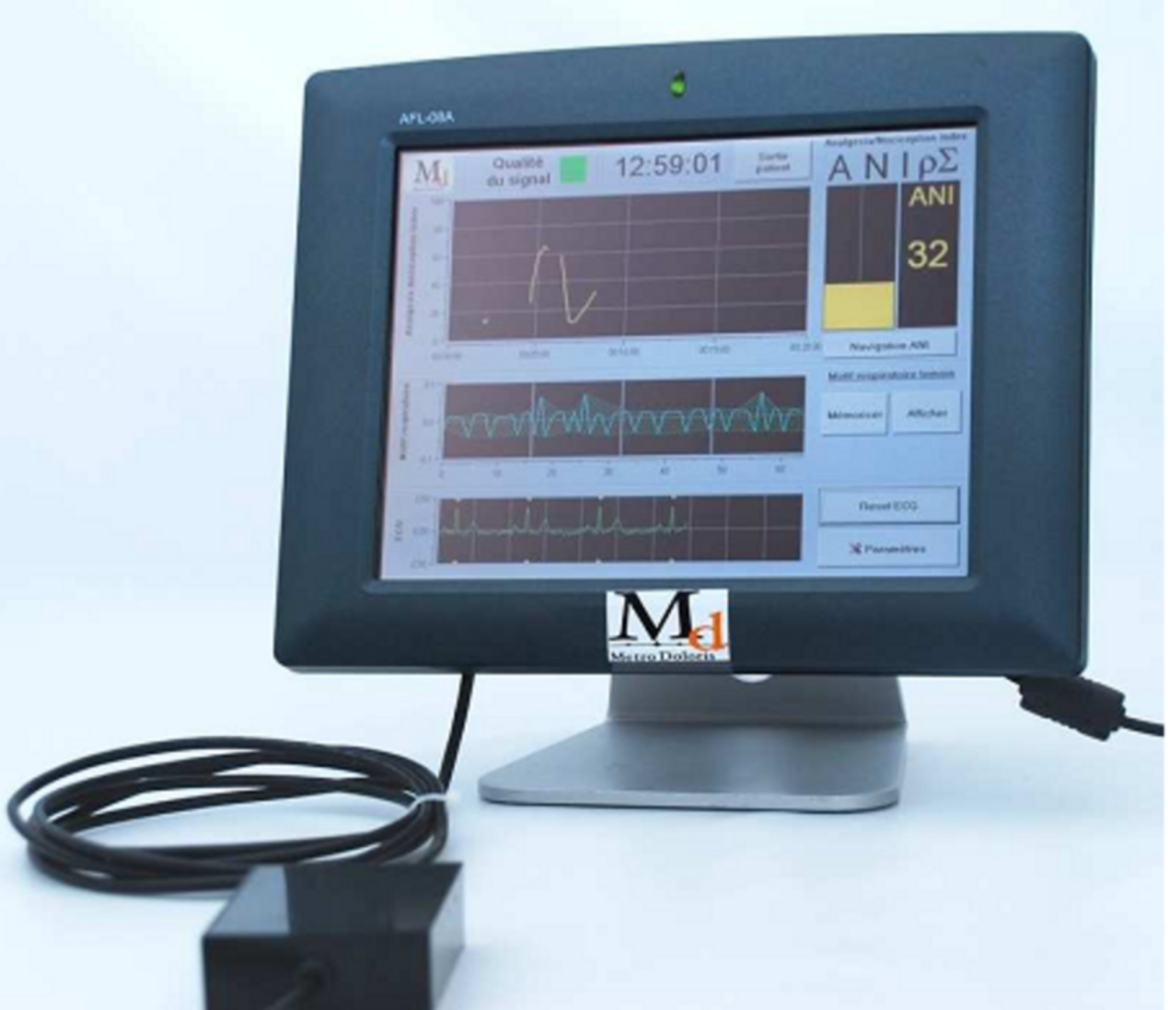
Physiodoloris monitor Lower scope: ECG signal, middle scope: normalized, mean centered and band-pass-filtered RR series, upper scope: ANI trend curve ECG, electrocardiography; ANI, analgesia nociception index

The Physiodoloris® monitor (Figure 2) commercialized by MetroDoloris® (Lille, France) is used to measure the abovementioned parameter.

**Figure 3:**
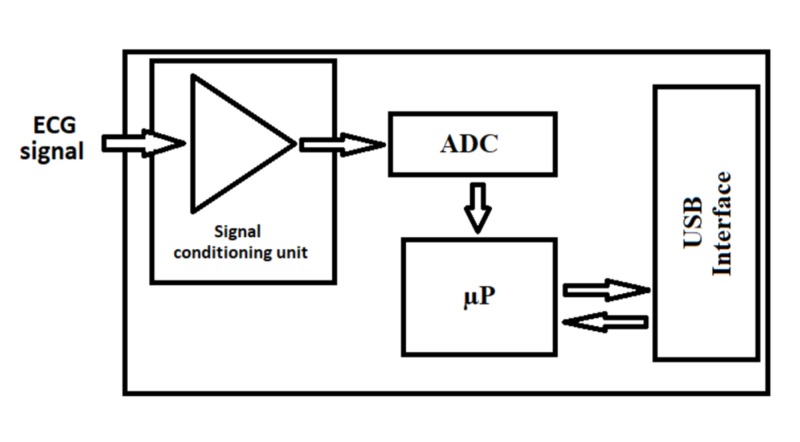
Electronic module with an ECG amplifier, ADC and computing capabilities and a USB communication port ECG, electrocardiography; ADC, analog-to-digital converter; USB, universal serial bus

**Figure 4:**
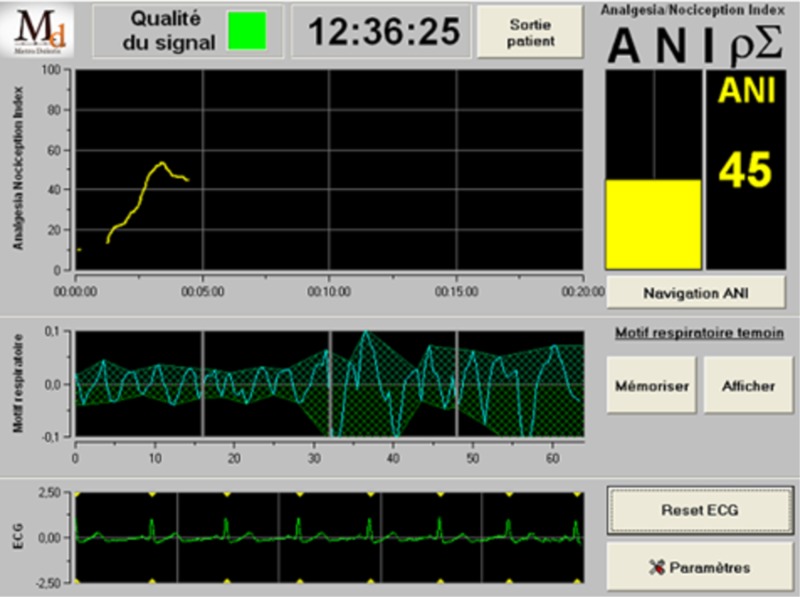
PC screen displaying signals and parameters PC, personal computer

The monitoring device is composed of an acquisition and treatment module (Figure 3), and a software part implemented on a classical personal computer in charge of display and human/machine interface (Figure 4).

The whole process of ECG acquisition, R wave detection, R-R series filtering by wavelet transform, normalization and ANI computation is implemented in a microcontroller. ANI measurements are transmitted by the microcontroller through the USB communication interface. The software part implemented of the PC is just in charge of data display. A specific communication protocol allows transmitting the results in real time.

C. Clinical trial

After institutional approval (Malatya Klinik Araştırmalar Etik Kurulu, 08.06.2016, 2016/122), 20 adult volunteers were recruited for this prospective, observational experimental study, which evaluated ANI response to the emotional stimulus. Participants were anesthesia students at the Adiyaman University Research and Educational Hospital, and written informed consent was obtained from them. Participants with mental or somatic diseases were not included.

The measurements were obtained between 1 and 3 pm to avoid any possible circadian rhythm interactions. Test subjects were taken into the measurement room, where they spent 3 minutes to calm down and suppress possible excitement before the test. The environment was kept silent and doors closed to eliminate external interferences. A senior female anesthesia technician accompanied the test subjects to prevent any possible discomfort from the students' point of view. An emotional stimulus was obtained through a 60-second music sound record from the song “Ala Gözlerini Sevdiğim Dilber”, performed by the Turkish rock band Badem; the popular band, being loved by most. ANI measurements were obtained before the song presentation (Tpre), at the end of the record presentation (T0), and each minute thereafter until the end of the five-minute observation (T1-T5). At the end of the test, the participants were asked to assess their subjective emotional experience about the song on a 5-point Likert scale ranging from 1 (very bad) to 5 (very good).

D. Statistical analysis

Data analysis was performed using SPSS 15.0 (Statistical Package for the Social Sciences, Chicago, Illinois). Data were presented as the number of cases, mean, standard deviation (SD), median with minimum and maximum. The difference between six ANI measures was tested using the Friedman test with Bonferroni-adjusted Wilcoxon post hoc test. The statistical tests were considered significant at a *p-*value of 0.05.

## Results

A total of 20 participants were investigated; 10 males and 10 females. The mean age of the participants was 17.0 ± 0.9 (min: 16, max: 20).

**Figure 5:**
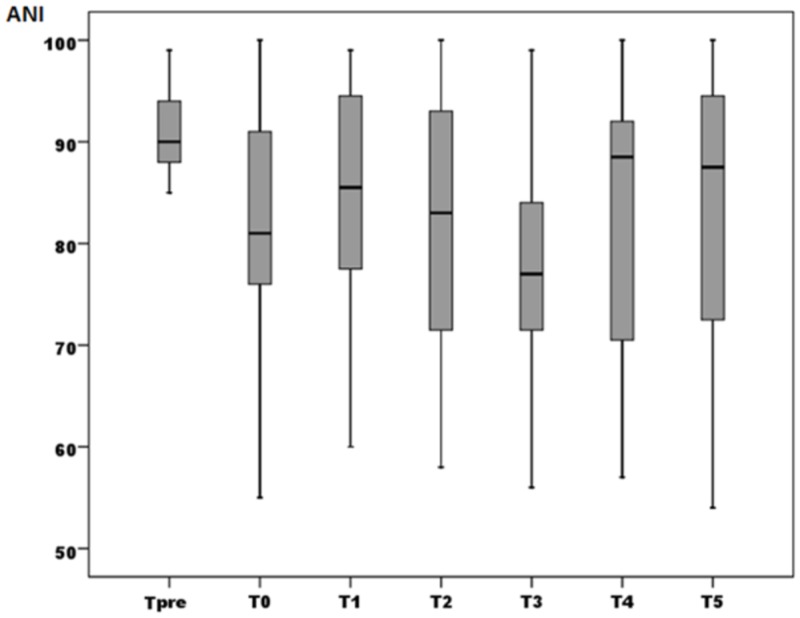
Box plot for ANI values ANI, analgesia nociception index; T_pre_, before the record presentation; T0, just after the presentation; T1-T5, at 1^st^-5^th^ minutes after the presentation. *P* < 0.05 for T0 and T3 compared with Tpre

ANI measurements were significantly lower in T0 and T3 compared with Tpre (*P* = 0.009). The differences between other values were not statistically significant (Table 1 and Figure 5).

**Table 1:** ANI values at Tpre, T0, and T1-5 ANI, analgesia nociception index; for T_pre_, T0-T5, see the text; *P < 0.05 for T0 and T3 compared with T_pre_

Time	Mean (SD)	Median (min-max)	P
T_pre_	90.1 (5.9)	90.0 (76-99)	0.009*
T0	81.8 (11.4)	81.0 (55-100)	
T1	84.1 (11.9)	85.5 (60-99)	
T2	82.0 (12.9)	83.0 (58-100)	
T3	78.1 (11.0)	77.0 (56-99)	
T4	82.6 (12.9)	88.5 (57-100)	
T5	83.9 (13.7)	87.5 (54-100)	

**Figure 6:**
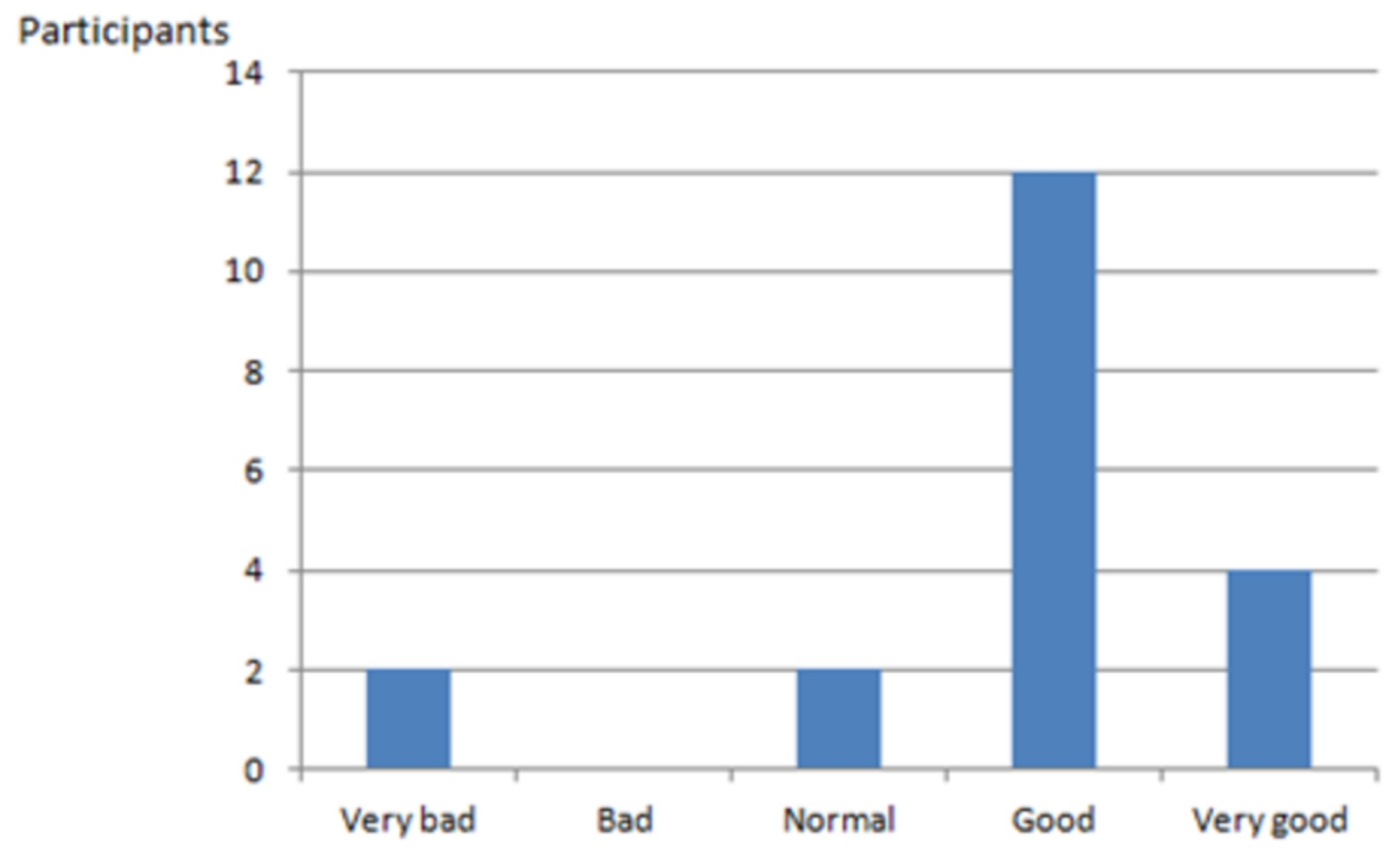
Music evaluation according to participants

The participants’ opinion about the presented music record, measured by a 5-point Likert scale is presented in Figure 6. Eighty percent of the participants credited the song as being good or very good.

## Discussion

In this study, we present the effects of emotional stimulation on ANS, which was evaluated by HRV analysis. Previously De jonckheere et al. have presented the application of HRV index in the evaluation of ANS changes related to a negative emotional stimulus generated by visual input [13]. Our study represents this domain of ANS evaluation from another perspective, using an auditory input.

The main result of the study is that an emotional stimulus is associated with an immediate decrease in ANI and that ANI monitoring is capable of measuring a change in the emotional status of a healthy volunteer. This result was consistent with the findings of De jonckheere et al. [13]. What was not consistent, is the second decline in ANI after three minutes of the stimulus cessation. De jonckheere et al. have found an immediate post-stimulus ANI decline and return to baseline in two minutes, whereas we demonstrated a second decline at 3rd minute. Looking at Figure 5, we can say that the measurements at two and four minutes are similar to the De jonckheere et al. measurements. The value at three minutes that is not concordant, and this measurement might have been spared in that study, because of the measurement intervals. In our study, the measurements were done in one minute apart.

Another reason for the second decline is the baseline variation of ANI scores. ANI was designed to make measurements in patients under general anesthesia, where other confounders of ANS like different levels of consciousness, external visual and auditory stimuli are absent. Hence, this might be not the best modality for analgesia measurement in conscious people, where external visual and auditory stimuli or even thoughts present at the measurement time cannot be totally eliminated.

Optimization of analgesia requires pain assessment of the patient. In conscious patients, subjective pain scores like visual analog scale (VAS), numerical rating scale (NRS), or faces pain scale (FPS) are used. On the other hand, heart rate (HR) and blood pressure (BP) are used for pain assessment under general anesthesia. However, these parameters are neither sensitive nor specific for pain and analgesia and are affected by multiple factors like drugs, comorbidities and volume status of the patient. ANI was recently introduced, measuring the parasympathetic tone based on HRV. As the bispectral index or the spectral entropy gives information about the hypnotic component of general anesthesia, ANI gives the information about the analgesic component of general anesthesia [17]. Pain, stress, anxiety or fear result in a decrease of the heart rate HF content [6,7,18]. ANI has been shown to detect noxious stimulus earlier and better compared with the above-mentioned hemodynamic parameters [11,16,19-20]. There are conflicting reports about the ANI of predicting hemodynamic changes [21-22].

Surgical Pleth Index (SPI), skin conductance, and pupillary reflex dilatation have also been used to predict nociception under general anesthesia; and ANI was at least as good as these modalities in this manner [23-25]. ANI was also used in conscious patients to assess antinociception/nociception balance during and after the surgery [26-27].

The main limitation of the study was that the volunteers were conscious. ANI was designed to make measurements in unconscious patients, because of many factors influencing ANS in conscious ones. Although optimal conditions were provided in the measurement room, some of the subjects were noted to get bored during the measurement period, and this might have influenced the results. Another limitation is that this is the preliminary study, and the results cannot be generalized to all ages, and patients.

## Conclusions

In conclusion, ANI can be used for the assessment of parasympathetic changes related to the emotional state of conscious patients. Further large-scale experimental and clinical studies are needed to assess this effect.

## References

[1] Friedman BH, Thayer JF (1998). Autonomic balance revisited: panic anxiety and heart rate variability. J Psychosom Res.

[2] Porges SW (1997). Emotion: an evolutionary by-product of the neural regulation of the autonomic nervous system. Ann N Y Acad Sci.

[3] Porges SW (2001). The polyvagal theory: phylogenetic substrates of a social nervous system. Int J Psychophysiol.

[4] Saul JP, Berger RD, Albrecht P, Stein SP, Chen MH, Cohen RJ (1991). Transferfunction analysis of the circulation: unique insights into cardiovascular regulation. Am J Physiol.

[5] Parati G, Mancia G, Di Rienzo M, Castiglioni P (2006). Point: cardiovascular variability is/is not an index of autonomic control of circulation. J Appl Physiol.

[6] Miu AC, Heilman RM, Miclea M (2009). Reduced heart rate variability and vagal tone in anxiety: trait versus state, and the effects of autogenic training. Auton Neurosci.

[7] Demaree HA, Robinson JL, Everhart DE, Schmeichel BJ (2004). Resting RSA is associated with natural and self-regulated responses to negative emotional stimuli. Brain Cogn.

[8] Appelhans BM, Luecken LJ (2008). Heart rate variability and pain: associations of two interrelated homeostatic processes. Psychol.

[9] Pan RL, Li JK (2007). A Noninvasive parametric evaluation of stress effects on global cardiovascular function. Cardiovasc Eng.

[10] Dishman RK, Nakamura Y, Garcia ME, Thompson RW, Dunn AL, Blair SN (2000). Heart rate variability, trait anxiety, and perceived stress among physically fit men and women. Int J Psychophysiol.

[11] Jeanne M, Logier R, De Jonckheere J, Tavernier B (2009). Validation of a graphic measurement of heart rate variability to assess analgesia/nociception balance during general anesthesia. IEEE Eng Med Biol Soc.

[12] Logier R, Jeanne M, Tavernier B (2004). Method and device for assessing pain in human being. University Hospital of Lille, University of Lille II.

[13] De jonckheere J, Rommel D, Nandrino JL, Jeanne M, Logier R (2012). Heart rate variability analysis as an index of emotion regulation processes: interest of the analgesia nociception index (ANI). IEEE Eng Med Biol Soc.

[14] Logier R, Gehin AL, Bayard M (1993, pp). Detection of transient myocardial ischemia by numerical processing of the electrocardiographique signal. IMACS International Symposium on Signal Processing, Robotics and Neural Networks.

[15] Logier R, De Jonckheere J, Dassonneville A (2004). An efficient algorithm for R-R intervals series filtering. IEEE Eng Med Biol Soc.

[16] Logier R, Jeanne M, De jonckheere J, Dassonneville A, Delecroix M, Tavernier B (2010). PhysioDoloris: a monitoring device for analgesia/nociception balance evaluation using heart rate variability analysis. IEEE Eng Med Biol Soc.

[17] De jonckheere J, Delecroix M, Jeanne M, Keribedj A, Couturier N, Logier R (2013). Automated analgesic drugs delivery guided by vagal tone evaluation: interest of the analgesia nociception index (ANI). IEEE Eng Med Biol Soc.

[18] Appelhans BM, Luecken LJ (2008). Heart rate variability and pain: associations of two interrelated homeostatic processes. Psychol.

[19] Jeanne M, Clément C, De Jonckheere J, Logier R, Tavernier B (2012). Variations of the analgesia nociception index during general anaesthesia for laparoscopic abdominal surgery. J Clin Monit Comput.

[20] Logier R, De Jonckheere J, Delecroix M, Keribedj A, Jeanne M, Jounwaz R, Tavernier B (2011). Heart rate variability analysis for arterial hypertension etiological diagnosis during surgical procedures under tourniquet. IEEE Eng Med Biol Soc.

[21] Ledowski T, Averhoff L, Tiong WS, Lee C (2014). Analgesia nociception index (ANI) to predict intraoperative haemodynamic changes: results of a pilot investigation. Acta Anaesthesiol Scand.

[22] Boselli E, Bouvet L, Bégou G, Torkmani S, Allaouchiche B (2015). Prediction of haemodynamic reactivity during total intravenous anaesthesia for suspension laryngoscopy using analgesia/nociception index (ANI): a prospective observational study. Minerva Anestesiol.

[23] Gruenewald M, Herz J, Schoenherr T, Thee C, Steinfath M, Bein B (2015). Measurement of the nociceptive balance by analgesia nociception index (ANI) and surgical pleth index (SPI) during sevoflurane - remifentanil anaesthesia. Minerva Anestesiol.

[24] Migeon A, Desgranges FP, Chassard D (2013). Pupillary reflex dilatation and analgesia nociception index monitoring to assess the effectiveness of regional anesthesia in children anesthetised with sevoflurane. Paediatr Anaesth.

[25] Sabourdin N, Arnaout M, Louvet N, Guye ML, Piana F, Constant I (2013). Pain monitoring in anesthetized children: first assessment of skin conductance and analgesia-nociception index at different infusion rates of remifentanil. Paediatr Anaesth.

[26] Jeanne M, Delecroix M, De Jonckheere J, Keribedj A, Logier R, Tavernier B (2014). Variations of the analgesia nociception index during propofol anesthesia for total knee replacement. Clin J Pain.

[27] Treister R, Kliger M, Zuckerman G, Goor Aryeh I, Eisenberg E (2012). Differentiating between heat pain intensities: the combined effect of multiple autonomic parameters. Pain.

